# Instrument-assisted cross fiber massage increases tissue perfusion and alters microvascular morphology in the vicinity of healing knee ligaments

**DOI:** 10.1186/1472-6882-13-240

**Published:** 2013-09-28

**Authors:** M Terry Loghmani, Stuart J Warden

**Affiliations:** 1 Department of Physical Therapy, School of Health and Rehabilitation Sciences, Indiana University, 1140 W. Michigan Street, CF-326, Indianapolis 46202, IN, USA; 2 Center for Translational Musculoskeletal Research, School of Health and Rehabilitation Sciences, Indiana University, 1140 W. Michigan Street, Indianapolis 46202, IN, USA

**Keywords:** Angiogenesis, Blood flow, Knee, Laser doppler imaging, Ligament, Massage

## Abstract

**Background:**

Ligament injuries are common clinical problems for which there are few established interventions. Instrument-assisted cross fiber massage (IACFM) was recently shown to accelerate the restoration of biomechanical properties in injured rodent knee medial collateral ligaments (MCL). The current study aimed to investigate the influence of IACFM on regional perfusion and vascularity in the vicinity of healing rodent knee MCL injuries.

**Methods:**

Bilateral knee MCL injuries were induced in female Sprague–Dawley rats. Commencing 1 week post-injury, 1 minute of IACFM was introduced unilaterally 3 times/week for 3 weeks. The contralateral injured MCL served as an internal control. Regional tissue perfusion was assessed *in vivo* throughout healing using laser Doppler imaging, whereas regional microvascular morphology was assessed *ex vivo* via micro-computed tomography of vessels filled with contrast.

**Results:**

IACFM had no effect on tissue perfusion when assessed immediately, or at 5, 10, 15 or 20 min following intervention (all *p* > 0.05). However, IACFM-treated hindlimbs had enhanced tissue perfusion when assessed 1 day following the 4th and 9th (last) treatment sessions (all *p* < 0.05). IACFM-treated hindlimbs also had greater perfusion when assessed 1 wk following the final treatment session (32 days post-injury) (*p* < 0.05). Subsequent investigation of microvascular morphology found IACFM to increase the proportion of arteriole-sized blood vessels (5.9 to <41.2 μm) in the tibial third of the ligament (*p* < 0.05).

**Conclusions:**

These findings suggest IACFM alters regional perfusion and vascularity in the vicinity of healing rodent knee MCL injuries. This effect may contribute to the beneficial effect of IACFM observed on the recovery of knee ligament biomechanical properties following injury.

## Background

Ligament injuries are prevalent accounting for approximately 50% of athletic injuries [1]. The majority of injuries are to capsular and extracapsular ligaments (such as the knee and ankle collateral ligaments), with the resultant imbalances in joint mobility and stability leading acutely to pain and functional limitations, and chronically to disability, permanent joint dysfunction, susceptibility to re-injury, and ultimately joint disease [2-5]. It is well established that surgery is not indicated for most capsular and extracapsular ligament injuries, including both partial and full thickness tears [6-8]. Consequently, there is a need to establish alternative interventions that facilitate recovery and enhance outcomes following ligament injury.

Numerous preclinical studies have investigated the utility of novel interventions targeting ligament healing, including the use of gene therapies, growth factors, biological scaffolds, stem cell therapies, and biophysical modalities [9-13]. Each of these directions has shown promise in influencing ligament healing; however, the techniques are far from being translated into the clinical realm and their eventual costs may prohibit wide use in mainstream clinical practice. Manual therapy represents an alternative intervention for influencing ligament healing. Manual therapy involves the application of specifically directed forces to the body in order to induce physiological and/or structural tissue changes. One of the oldest and most frequent forms of manual therapy is massage, with a popular rehabilitative technique being cross fiber massage.

Cross-fiber massage (also known as deep transverse or friction massage) involves applying the finger/s or a rigid instrument directly over a tissue lesion and transverse to the direction of the underlying collagen fibers. It was developed in an empirical way by Cyriax [14]; however, was first described by Hippocrates. The later used ‘anatripsis’ (Greek for friction) to treat ligament sprains and joint dislocations stating that ‘…hard rubbing binds…much rubbing causes parts to waste…and moderate rubbing makes them grow’ [15]. Despite its lengthy history, there is minimal scientific evidence of the benefits of cross fiber massage.

We recently performed a study investigating the benefit of a specific form of cross fiber massage, instrument assisted cross fiber massage (IACFM), following knee medial collateral ligament (MCL) injury in an established animal model [16]. IACFM utilizes a specially designed, hand-held instrument made out of a rigid material (i.e. stainless steel) to administer localized mechanical forces to subcutaneous or deep connective tissue structures. Findings in our previous study indicated that IACFM accelerated the return of tissue-level biomechanical properties in the healing ligaments when assessed at four weeks post-injury [16].

The acceleration of biomechanical healing with IACFM may be due to transduction of the mechanical stimulus into a collagen-synthesis response by fibroblasts, with previous studies suggesting IACFM facilitated the recruitment and activation of fibroblasts and altered the size and density of collagen fibers in rodent tendons [17-19]. However, it is possible that IACFM also influences vascular responses during ligament healing. Each phase of ligament healing requires adequate blood supply for the transport of cells and metabolites [20], and the influence of IACFM on vascular responses during ligament healing has not previously been explored. The purpose of the current study was to investigate the effect of IACFM on vascular properties (tissue perfusion and microvasculature morphology) in the vicinity of healing rodent knee MCL injuries.

## Methods

### Animals

Thirty adult, virgin, female, Sprague–Dawley rats (age: 6 months; weight: 280–300 g) were purchased from Harlan Spraque-Dawley, Inc. (Indianapolis, IN) and acclimated for a minimum of 7 days prior to experimentation. Animals were housed in standardized conditions with *ad libitum* access to standard rat chow and water at all times. All procedures were approved *a priori* by the Institutional Animal Care and Use Committee of Indiana University.

### Ligament injury

All animals underwent surgery on entry to the study to create bilateral knee MCL injuries, as previously described [16]. Following a subcutaneous dose of pre-operative analgesia (buprenorphine hydrochloride [0.05 mg/kg]; Reckitt & Colman Pharmaceuticals Ltd., Richmond, VA), surgical anesthesia was achieved via an intraperitoneal injection of ketamine (60–80 mg/kg; Fort Dodge Animal Health, Fort Dodge, IA) and xylazine (7.5 mg/kg; Fort Dodge Animal Health, Fort Dodge, IA). A 5-mm longitudinal incision was made over one knee medial joint line, and the MCL located and sharply transected at the joint line using a size 11 scalpel blade. The result was a complete mid-substance disruption of the ligament transverse to its collagen fiber alignment. The ligament ends were juxtaposed, but not sutured and the skin incision closed using a single subcuticular absorbable suture. The procedure was repeated on the contralateral side to create bilateral injuries.

### IACFM intervention

**Figure 1:**
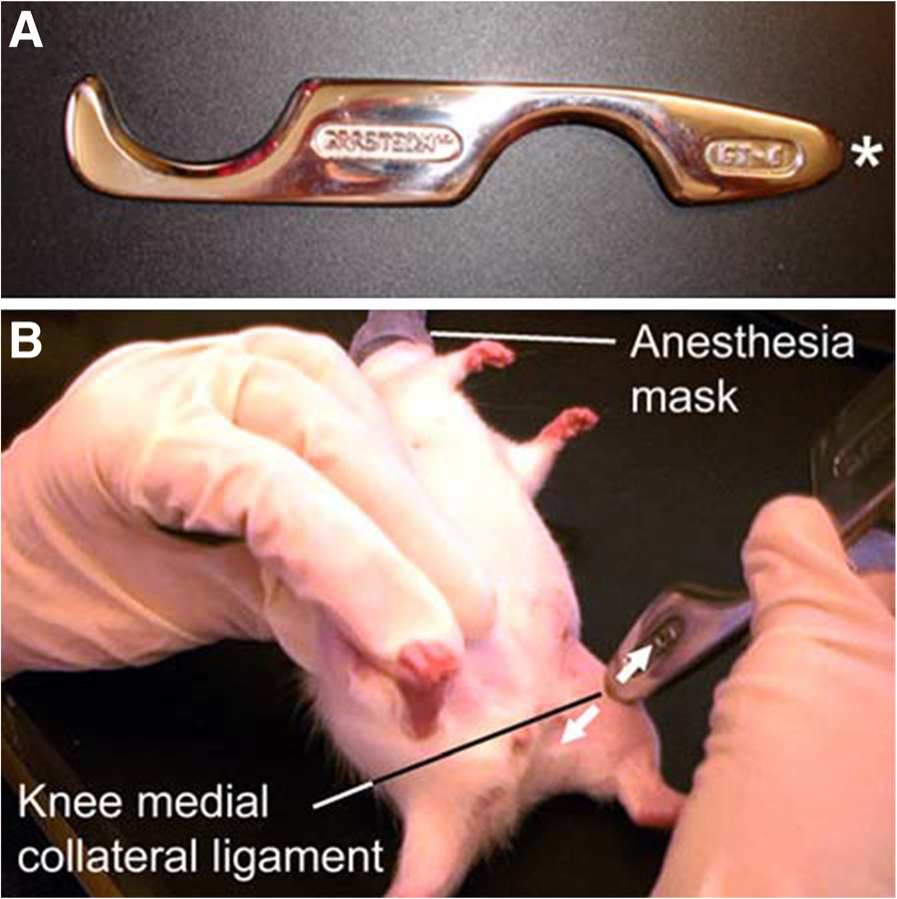
**Instrument-assisted cross fiber massage (IACFM) intervention. A**: The rigid Graston Technique® GT6 tool fabricated from stainless steel has a tapered tip (*) which permits treatment of small structures. **B**: IACFM of the rodent knee joint medial collateral ligament using the GT6 tool. Arrows indicate the direction of movement/force application perpendicular to the collagen substructure of the ligament.

IACFM was delivered using a rigid tool fabricated from stainless steel (Graston Technique®, TherapyCare Resources, Indianapolis, IN) (Figure 1A). The protocol was based that described in similar studies [16-18]. IACFM was initiated 1 wk following injury induction (post-acute) to allow completion of the initial inflammatory phase. Delaying the introduction of IACFM is consistent with its suggested clinical use following acute injury. IACFM was delivered for one minute to the left injured MCL (IACFM-treated) with the animals under inhalation anesthesia (Figure 1B). The delivery time was based on prior animal model studies indicating the efficacy of short duration of IACFM applications [16-18]. Approximately 250–300 g of downward force was applied during the treatment, as determined by using the massage instrument on a force plate with a force equivalent to that employed clinically. This amount of force also corresponds with previous studies demonstrating the benefit of IACFM during tendon and ligament healing in rats [16-18]. Animals were treated 3 times/wk for 3 wk (total treatment sessions = 9). The contralateral, injured MCL was not treated and served as an internal control (non-treated).

### Assessment of superficial regional tissue perfusion

Regional superficial tissue perfusion was assessed *in vivo* in one set of animals (n = 11) using noninvasive laser Doppler imaging (LDI). A desktop scanning laser-Doppler perfusion imager (MoorLDI2-IR™; Moor Instruments, Wilmington, DE) was used, as per manufacturer guidelines. The imager possessed a laser diode source that produced near-infrared energy with a wavelength of 785 nm and nominal power of 2.25 mW. A low-frequency cut-off (250 Hz) was used to eliminate movement artifacts, while a high-frequency cut-off (15 kHz) was used to improve the signal-to-noise ratio.

LDI was performed in a standardized environment (22°C) with animals under inhalation anesthesia. The hindlimbs were shaved, and the animals were placed in dorsal recumbency on a warming pad (37°C) with both hindlimbs abducted to permit imaging of the medial knee joint region. Both hindlimbs were positioned within a single field of view to allow simultaneous scanning of the IACFM- and non-treated hindlimbs. The imager and animal were subsequently enclosed in an opaque screen to eliminate artifactual light during scanning. After animals were positioned, they were left undisturbed for 10 min to reduce handling and environmental influences on tissue perfusion measures.

Triplicate scans of each animal were performed without interim repositioning at the following time points: the day prior to surgically-induced MCL injury (pre-injury), immediately prior to each IACFM intervention (pre-IACFM), and at 0, 5, 10, 15 and 20 min, and 24 hr following each IACFM intervention (post-IACFM). A final assessment was performed 1 wk following the final IACFM intervention (4 wk post-injury). As the IACFM- and non-treated hindlimbs were simultaneously scanned within each animal, the influences of subtle changes in ambient temperature and light, and physiological status of the animals were controlled. An identical region of interest was selected over the medial knee region on the acquired 2D images, and mean unitless flux values (perfusion units) were obtained for both the IACFM- and non-treated hindlimbs. These flux values were averaged for the triplicate scans performed on each hindlimb at each assessment time point. The MCLs from these animals were harvested at 5 wk post-surgery, and assessed for structural and mechanical properties. These later data have previously been published [16].

### Assessment of regional microvasculature morphology

Microvasculature morphology was assessed in a second set of animals (n = 19) by micro-computed tomography (micro-CT) imaging of tissues perfused with a contrast agent, based on procedures described in the literature [21-23]. Animals were anesthetized at 4 wk post-surgery (1 wk following the final IACFM intervention) with an intraperitoneal injection of ketamine (60–80 mg/kg; Fort Dodge Animal Health, Fort Dodge, IA) and xylazine (7.5 mg/kg; Fort Dodge Animal Health, Fort Dodge, IA). They were placed on a warming pad (37°C) in dorsal recumbency with their appendages splayed and fixed. The thoracic cavity was opened, and the inferior vena cava located and severed to exsanguinate the animals. A 16-G cannula needle connected to a perfusion pump (Minipuls 2 Peristaltic Pump; Gilson Inc., Middleton, WI) was inserted through the left ventricle of the heart and into the ascending aorta. The vasculature was flushed with 0.9% normal saline containing heparin sodium (100U/ml) and sodium nitrite before being pressure fixed with 4% phosphate-buffered formalin. Formalin was flushed using heparinized saline and the vasculature perfused with a radiopaque silicone rubber containing lead chromate (Microfil MV-122, Flow Tech, Inc., Carver, MA). Following storage at 4°C overnight to allow contrast agent polymerization, the hindlimbs were removed and soaked in 10% neutral buffered formalin for 4 days to ensure complete tissue fixation.

Immediately prior to micro-CT imaging, the MCLs and their adjacent connective tissues were harvested and dissected to a standard sample size (length = 1.5 cm, width = 0.5 cm). Adjacent connective tissue was included in analyses as the MCL proper has limited vascularity, and receives its blood supply from vessels in its epiligament and the adjacent periarticular tissues [24]. This approach maximized the number of vessels assessed, with the tissues containing these vessels also being injured during surgery and subsequently treated during IACFM intervention. Samples were positioned vertically on the computer-controlled rotation stage of a bench top micro-CT system (SkyScan 1172 high-resolution micro-CT; SkyScan, Kontich, Belgium) and scanned 180° around the vertical axis in rotation steps of 0.4° using an x-ray source operating at 50kV. The isotropic voxel size was 5.9 μm. Serial tomograms were reconstructed and thresholded to segment the radiopaque microvasculature network from its surrounding connective tissue. This was initially performed for the whole tissue sample before the tomograms were subdivided into thirds corresponding with the femoral, middle and tibial portions of the specimen. Microvasculature morphology parameters acquired from the whole tissue, and femoral, middle and tibial sub-regional analyses included: vessel volume normalized to tissue volume (VV/TV; %), vessel number (V.N;/mm), vessel thickness or diameter (V.Th; μm), and vessel separation (V.Sp; mm). In addition, the frequency distribution of V.Th in each femoral, middle and tibial sub-region was assessed.

### Statistical analyses

LDI flux values in IACFM-treated hindlimbs were expressed relative to those measured in non-treated hindlimbs (IACFM treated/non-treated) to provide a perfusion ratio for each individual animal. Intervention effects were assessed at each time point by calculating mean perfusion ratios and their 95% confidence intervals (CI). 95% CI not crossing 1 were considered statistically significant, as determined by single sample t-tests on the mean perfusion ratios with a population mean of 1. Intervention effects on regional microvasculature morphology measures were assessed using paired t-tests. All comparisons were two-tailed with a level of significance set at 0.05.

## Results

### Regional tissue perfusion

**Figure 2:**
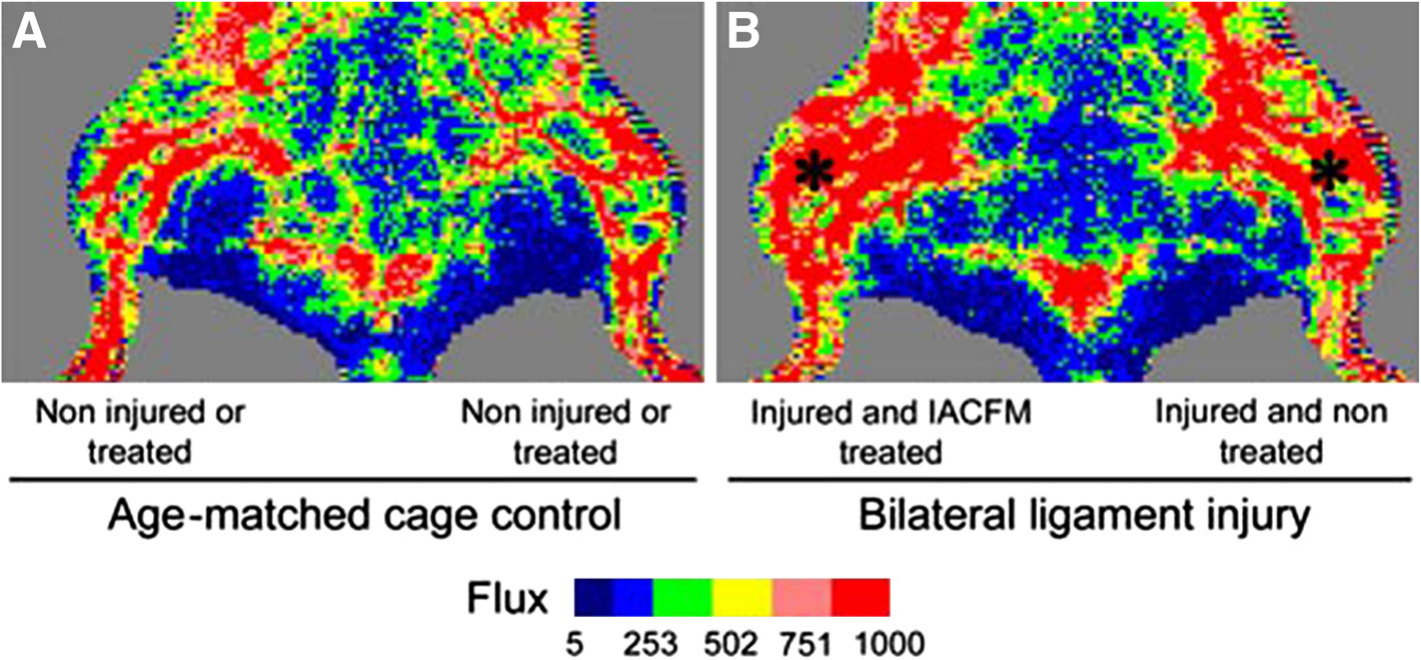
Representative laser Doppler images for the left and right hindlimbs in A: aged-matched uninjured control animal and B: animal with bilateral knee MCL injuries (*) at four weeks post-injury. A: Tissue flux (perfusion) was equivalent in the right and left legs of age-matched control animals, animals prior to surgically-induced MCL injury, and animals immediately prior to the initial IACFM treatment (i.e. one week following MCL injury). **B**: Tissue flux (perfusion) was increased in the IACFM treated hindlimb at 4 weeks following injury and 1 week following the final IACFM treatment, compared to the contralateral non-treated injured hindlimb.

**Figure 3:**
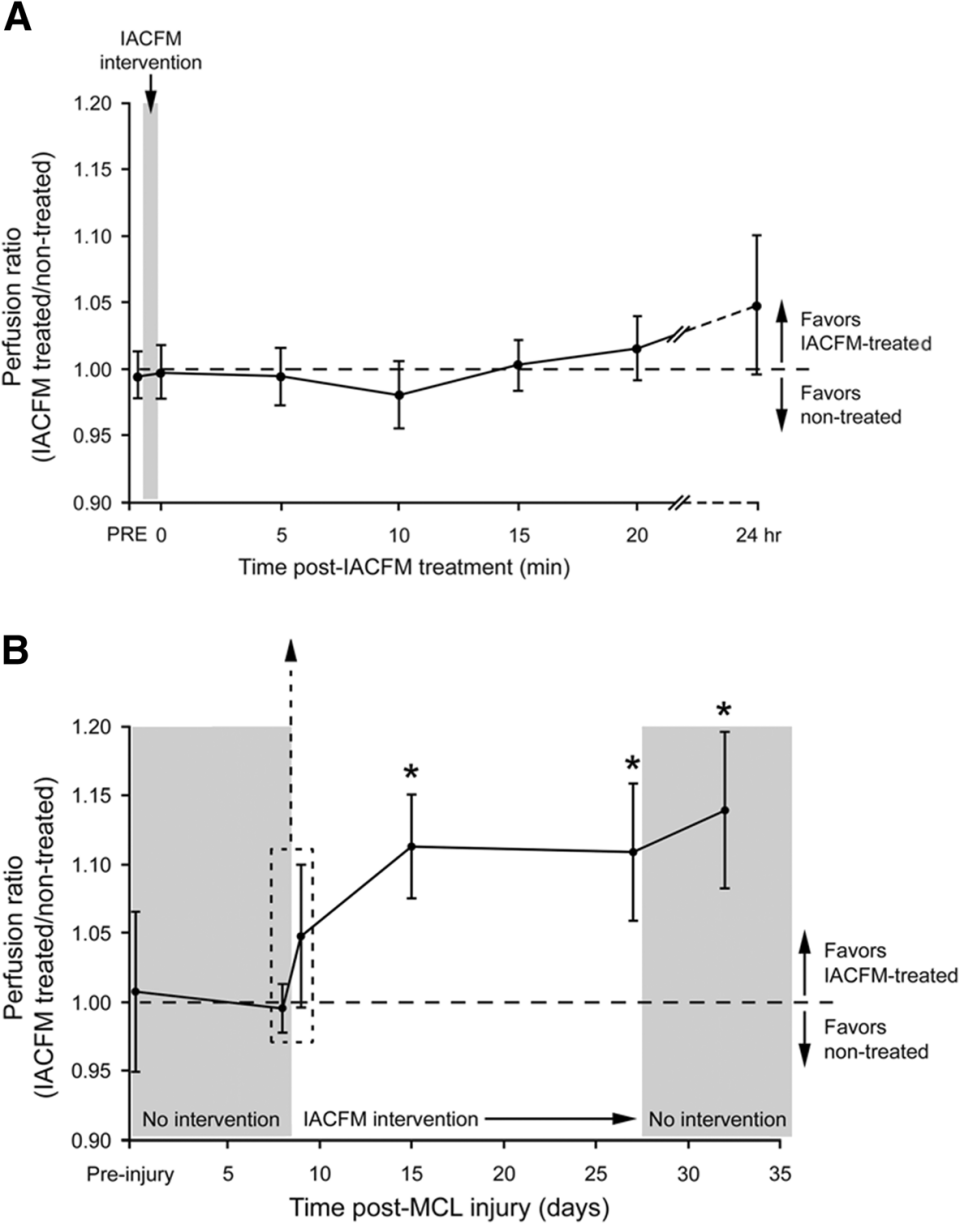
**Effect of IACFM on regional tissue perfusion following knee MCL injury. A**: IACFM had no immediate effect on tissue perfusion ratios, with ratios immediately prior (PRE) and after (0) the first IACFM treatment, at 5, 10, 15 and 20 minutes post-treatment, and at 24 hours post-treatment not differing significantly from 1 (i.e. perfusion in IACFM-treated = perfusion in non-treated). **B**: IACFM significantly increased tissue perfusion over time. In addition to having increased perfusion when assessed at 24 hours after the 4th and last (9th) treatment (15 and 26 days post-MCL injury, respectively), IACFM treated hindlimbs had significantly greater perfusion when assessed one week following the final treatment session (32 days post-MCL injury). Data represent perfusion ratios (IACFM treated/non treated) between IACFM treated and non-treated hindlimbs, with error bars indicating the 95% confidence interval. Ratios >1 indicate greater perfusion in IACFM-treated hindlimbs. *p < 0.05, as determined by single sample t-tests with a population mean of 1.

Representative laser Doppler images in an uninjured control animal and animal with bilateral knee MCL injuries at four weeks post-injury are shown in Figure 2. There were no side-to-side differences in regional tissue blood flow in animals prior to surgically-induced MCL injury or immediately prior to the initial IACFM intervention (all *p* > 0.05) (Figure 3). There was no immediate effect of IACFM on tissue perfusion, with IACFM- and non-treated hindlimbs having equivalent perfusion immediately, and at 5, 10, 15 and 20 min following the IACFM treatment session (all *p* > 0.05) (Figure 3A). However, when assessed 24 hr following the 4th and 9th (last) treatment sessions (15 and 26 days post-injury, respectively) IACFM-treated hindlimbs had significantly greater tissue perfusion than contralateral non-treated hindlimbs (all *p* < 0.05) (Figure 3B). Also, IACFM-treated hindlimbs had significantly greater perfusion when assessed 1 wk following the final treatment session (32 days post-injury) (*p* < 0.05) (Figure 3B).

### Morphology of the microvasculature

**Figure 4:**
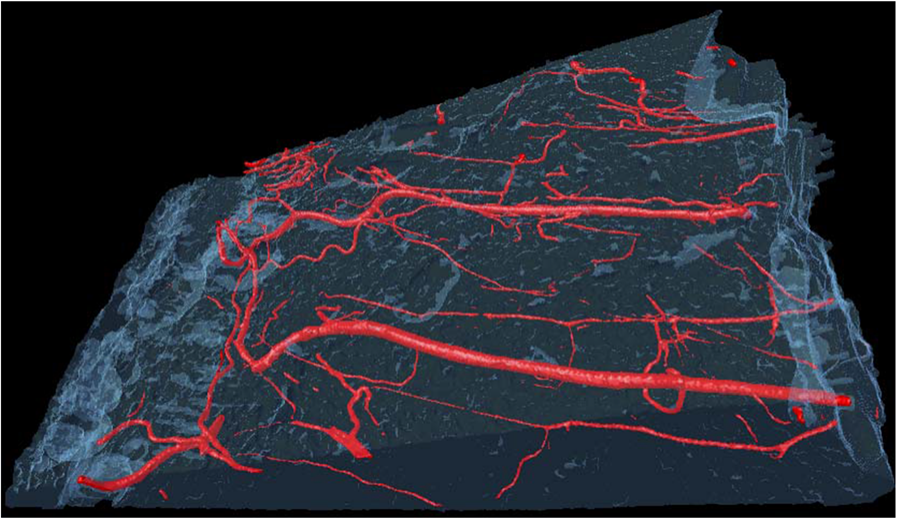
**Representative micro-CT image of contrast agent perfused blood vessels within a MCL and its surrounding connective tissue.** Microvasculature morphology parameters acquired from the whole tissue, and upper (femoral), middle and lower (tibial) thirds of the tissue volume were: vessel volume normalized to tissue volume (VV/TV; %), vessel number (V.N;/mm), vessel thickness or diameter (V.Th; μm), and vessel separation (V.Sp; mm). The MCL is not distinguishable within the tissue volume because of its equivalent radiopacity. Images from both IACFM-treated and non-treated hind limbs are not shown as they are visually indistinguishable.

**Figure 5:**
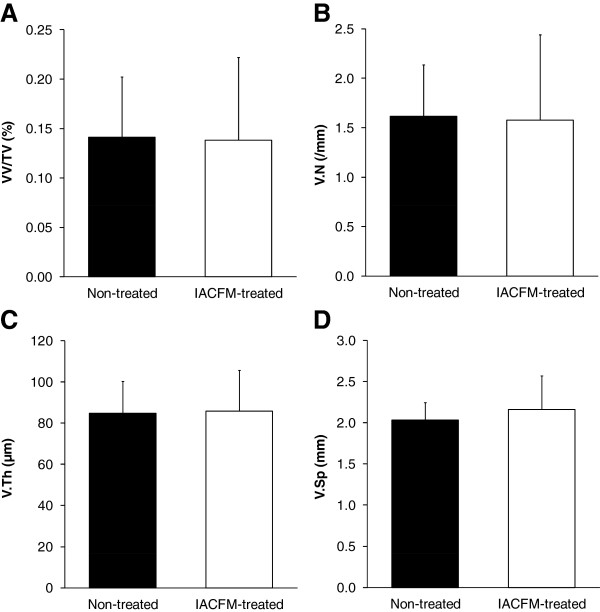
**Effect of IACFM on microvasculature morphometric parameters following knee MCL injury.** There were no significant IACFM effects on **A**: vessel volume normalized to tissue volume [VV/TV], **B**: vessel number [V.N], **C**: vessel thickness or diameter [V.Th], or **D**: vessel number [V.N] (all *p* = 0.37 to 0.90). Bars represent mean ± SD.

**Figure 6:**
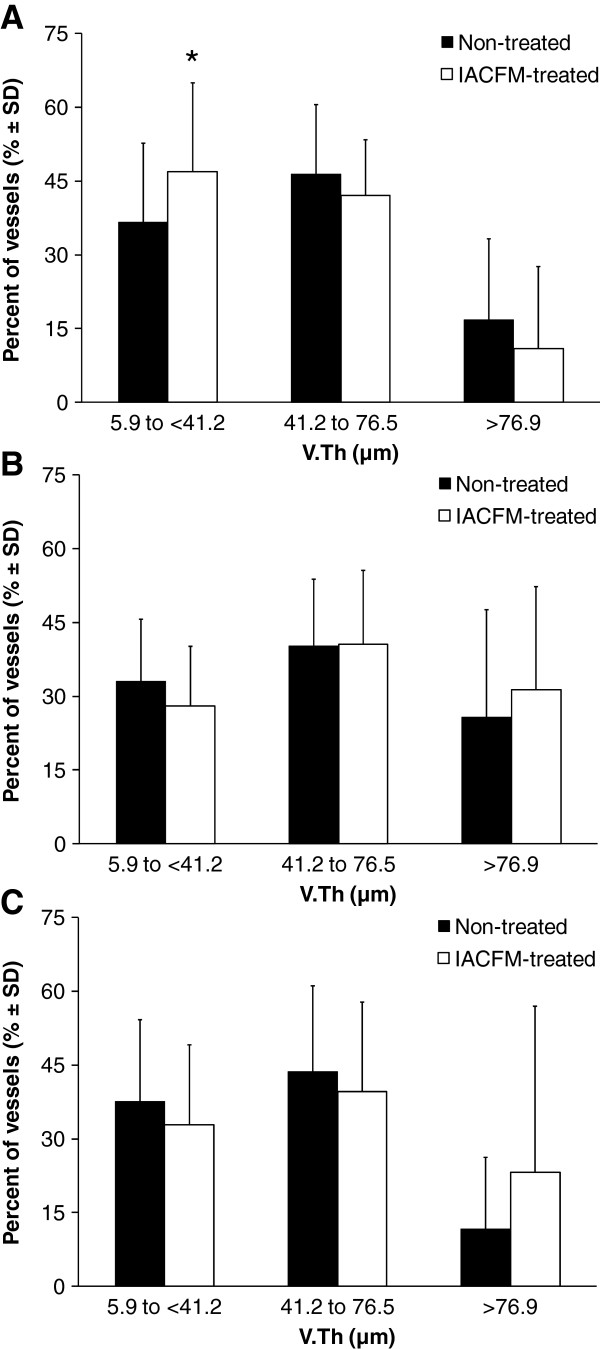
**Effect of IACFM on the frequency distribution of vessel thickness (V.Th).** V.Th distribution in the **A**: tibial, **B**: middle, and **C**: femoral subregions of the injured knee MCL and adjacent connective tissue. IACFM-treated hindlimbs had a higher proportion of V.Th within the 5.9 to <41.2 μm range in the tibial subregion. No differences were found in the middle or femoral subregions. Bars represent mean ± SD. *indicates *p* < 0.02 compared to non-treated hindlimb.

A representative micro-CT image of contrast agent perfused blood vessels within a MCL and its surrounding connective tissue is shown in Figure 4. There were no significant differences in VV/TV, V.Th, V.N or V.Sp between IACFM- and non-treated hindlimbs (all *p* = 0.37 to 0.90) (Figure 5). This was confirmed following subdivision of the tissue volume into femoral, middle and tibial thirds, with VV/TV, V.N, V.Th or V.Sp not differing between IACFM- and non-treated hindlimbs within any sub-region (all *p* > 0.05) (*data not shown*). Analysis of the distribution of V.Th in the tibial subregion revealed IACFM-treated hindlimbs to have a higher proportion of vessels within the 5.9 μm to <41.2 μm range compared to non-treated hindlimbs (*p* < 0.02) (Figure 6A). There were no differences in the distribution of V.Th between IACFM- and non-treated hindlimbs in the femoral and middle subregions (all *p* > 0.05) (Figure 6B and C).

## Discussion

The results of this study suggest that IACFM increases blood flow and alters microvascular morphology in the vicinity of healing knee ligaments. IACFM treatment of surgically-induced knee MCL injuries increased regional tissue perfusion, as assessed using LDI. The increase was not observed immediately following intervention suggesting IACFM does not lead to a direct increase in tissue perfusion due to vasodilation. In contrast, IACFM increased tissue perfusion days following treatment, an effect that persisted for 1 wk following the final IACFM intervention. Subsequent investigation found IACFM to have no effect on global microvascular morphology within the injured ligament and its surrounding connective tissue, as assessed by quantitative micro-CT imaging of vessels filled with a contrast agent. However, further analyses revealed one portion of the assessed tissue region to have a greater proportion of smaller diameter blood vessels (5.9 to <41.2 μm), suggesting IACFM treatment had a subtle, yet measurable influence on localized microvascular morphology. Overall, our findings suggest that IACFM altered regional vascular properties following ligament injury. Whether the detected vascular changes contribute to the beneficial effect of IACFM on the recovery of knee MCL biomechanical properties observed in a previous study [16] requires further exploration.

The importance of blood supply to knee ligament healing is well established. Each stage of ligament healing depends upon adequate vascularity and blood flow for the delivery and removal of cells and metabolic substrates at the injury site [20]. Numerous studies have associated variations in blood supply with alterations in knee MCL healing [25-28], and differences in blood supply are thought to contribute to the differential healing of MCL and anterior cruciate ligament injuries [29,30]. Massage therapy is often introduced with the intent of altering tissue blood flow; however, studies have generally been limited to the exploration of immediate, short-term effects [31-34]. The immediate effects of massage on blood flow are hypothesized to be as a result of a vasodilatory effect of the therapy on vessels within the treated region. To our knowledge, no study has reported an effect of massage on regional vascular properties, particularly at delayed time points (>24 hours) following massage completion.

The delayed effect of IACFM therapy in the current study on tissue perfusion is a novel finding and suggests this form of massage therapy had a morphological effect on the vascular system in the vicinity of the healing MCL, as opposed to an immediate, more temporary vasodilatory effect. This hypothesis was partially supported by quantitative micro-CT imaging of vessels filled with a contrast agent, which showed the tibial portion of the healing MCL and its adjacent connective tissue to have a larger proportion of blood vessels in the diameter range of arterioles. The finding of alterations in the proportion of arteriole-sized vessels is potentially important as these vessels regulate the flow of blood through the capillary beds they supply.

It is uncertain why expansion of arteriole-sized vessels was isolated to the distal portion of the ligament. More dense distributions of epiligamentous vessels are located near the bony insertions of the MCL [24]. It may be that neovascularization within the injured MCL originates from these vessels. Alternatively, it is possible that expansion of arteriole-sized vessels in the distal portion of the MCL resulted from intensifying of treatment pressures at this site, with the ligament being compressed during intervention between the rigid massage tool and underlying tibia. An earlier study demonstrated elevated IACFM pressures were associated with enhanced cellular responses [18].

The current study extends knowledge regarding IACFM effects during ligament healing; however, the data are not without limitations. Rats were studied for handling, housing and cost considerations; however, they have small and difficult to study ligaments relative to larger species. The injury model of surgically transecting the MCL produced highly reproducible injuries for comparative research purposes, but it does not replicate the strain-related injury mechanism that occurs clinically. LDI provided interesting data regarding delayed effects of IACFM, yet it possesses a limited depth of penetration with only 37% of incident energy remaining after 1 mm of penetration in skin [35]. Thus, it remains unanswered whether IACFM actually influenced perfusion within the ligament proper in the current study. Other investigators have opened the skin to enable LDI to provide a more specific assessment of MCL perfusion [36,37]. This approach was not implemented in the current study as we wanted to obtain non-invasive measures in the same animals over time. There are also limitations with the technique used to image vascular morphology, including potential incomplete filling and/or overdistension of vessels, and the inability of being able to distinguish between the arterial and venous vascular systems.

## Conclusions

The results of this study suggest that IACFM increases perfusion and alters microvascular morphology in the vicinity of healing knee ligaments. These effects may contribute to the accelerated post-injury recovery of knee ligament biomechanical properties observed in a previous study [16], and confirm the need for further investigations into the effects of IACFM. In particular, future studies need to consider the mechanism of action and molecular effects of IACFM, and explore the impact of dosage variables. Also, further studies are indicated to establish the efficacy of IACFM in clinical populations.

## Abbreviations

CI: Confidence interval; IACFM: Instrument-assisted cross fiber massage; LDI: Laser Doppler imaging; MCL: Medial collateral ligament; V.N: Vessel number; V.Sp: Vessel separation; V.Th: Vessel thickness or diameter; VV/TV: Vessel volume normalized to tissue volume.

## Competing interests

The authors declare that they have no competing interests.

## Authors’ contributions

MTL performed interventions and outcome measures; MTL and SJW conceived the project, participated in study design, induced ligament injuries and interpreted data. All authors wrote and provided feedback on drafts of the manuscript. All authors read and approved the final manuscript.

## Pre-publication history

The pre-publication history for this paper can be accessed here:

http://www.biomedcentral.com/1472-6882/13/240/prepub
